# Exogenous 24-Epibrassinolide Improves Low-Temperature Tolerance of Maize Seedlings by Influencing Sugar Signaling and Metabolism

**DOI:** 10.3390/ijms26020585

**Published:** 2025-01-11

**Authors:** Siqi Sun, Xiaoqiang Zhao, Zhenzhen Shi, Fuqiang He, Guoxiang Qi, Xin Li, Yining Niu, Wenqi Zhou

**Affiliations:** 1 State Key Laboratory of Aridland Crop Science, College of Agronomy, Gansu Agricultural University, Lanzhou 730070, China; sunsiqi1215@163.com (S.S.); shizz@gsau.edu.cn (Z.S.); hefq6125@163.com (F.H.); qigx1321@163.com (G.Q.); m18214360633@163.com (X.L.); niuyn@gsau.edu.cn (Y.N.); 2 Crop Research Institute, Gansu Academy of Agricultural Sciences, Lanzhou 730070, China; zhouwenqi850202@163.com

**Keywords:** sugar, maize, coleoptile, 24-epibrassinolide, RNA-sequencing, quantitative reverse transcriptase PCR

## Abstract

Low-temperature (LT) stress seriously affects the distribution, seedling survival, and grain yield of maize. At the seedling emergence stage, maize’s coleoptile is one of the most sensitive organs in sensing LT signaling and, in general, it can envelop young leaves to protect them from LT damage. In addition, brassinolides (BRs) have been shown to enhance LT tolerance from various species, but the effects of BRs on coleoptiles in maize seedlings under LT stress are unclear. Therefore, in this study, the pre-cultured coleoptiles of Zheng58 seedlings were treated with or without 2.0 μM 24-epibrassinolide (EBR) at 25 °C and 10 °C environments for five days to analyze their physiological and transcriptomic changes. Physiological analysis showed that a 10°C LT stress increased the content of glucose (0.43 mg g^−1^ FW), sucrose (0.45 mg g^−1^ FW), and starch (0.76 mg g^−1^ FW) of Zheng58 coleoptiles compared to a 25°C environment. After the coleoptiles were exposed to a 2.0 μM EBR application under 10°C temperature for five days, the contents of these three sugars continued to increase, and reached 2.68 mg g^−1^ FW, 4.64 mg g^−1^ FW, and 9.27 mg g^−1^ FW, respectively, indicating that sugar signaling and metabolism played key roles in regulating LT tolerance in the coleoptiles of maize seedlings. Meanwhile, a transcriptome analysis showed that 84 and 15 differentially expressed genes (DEGs) were enriched in the sucrose and starch metabolism and photosynthesis pathways, respectively, and multiple DEGs involved in these pathways were significantly up-regulated under LT stress and EBR stimulation. Further analysis speculated that the four DEGs responsible for sucrose-phosphate synthetase (SPS, i.e., *Zm00001d048979*, probable sucrose-phosphate synthase 5 and *Zm00001d012036*, sucrose-phosphate synthase 1), sucrose synthase (SUS, *Zm00001d029091*, sucrose synthase 2 and *Zm00001d029087*, sucrose synthase 4) were crucial nodes that could potentially link photosynthesis and other unknown pathways to form the complex interaction networks of maize LT tolerance. In conclusion, our findings provide new insights into the molecular mechanisms of exogenous EBR in enhancing LT tolerance of maize seedlings and identified potential candidate genes to be used for LT tolerance breeding in maize.

## 1. Introduction

Maize (*Zea mays* L.) is a main cereal crop with high nutritional value, its average annual production holds steady at 1070 million tons in the world [1]. It is well known that maize originates from the tropical and subtropical regions [2], thus, it is extremely sensitive to low-temperature (LT, 5–15 °C) stress throughout its whole life cycle, but especially during seed germination to seedling morphological establishment [3,4,5]. In recent years, the abnormal global climate has led to longer cold waves in the spring, which has caused obvious adverse effects on seed germination and seedling growth of crops [4,6]. Generally, temperatures below 15 °C affect seed germination, seedling growth, which can cause seedling yellowing, wilting, and even death, resulting in 20–30% yield losses in the northern spring maize region of China [7,8,9]. In addition, LT stress can also cause irreversible damage to photosynthetic organs, reduce light energy utilization, increase excessive accumulation of reactive oxygen species (ROS) [10], increase oxidative stress [11], change antioxidant defense enzyme activities [12], destroy hormone homeostasis [13], aggravate membrane lipid oxidation [14], and activate cold-induced gene expression [4,12,15]. When maize plants are exposed to early spring frost damage, an accumulation of osmotic substances increases to maintain cellular osmotic pressure [16].

It is well known that brassinolides (BRs), a class of novel plant hormones, play crucial roles in plant growth and development [17,18]. Notably, they have successfully been applied to agricultural practices to increase production and enhance plant resistance to various environmental stresses, including drought [17,19,20,21], acid/saline-alkali soils [22], and nutrient deficiency [23]. For example, 0.5 μM EBR application under drought [24], salt and alkali [25], and heavy metal [26,27,28] improved crop yield by effectively increasing chlorophyll formation, improving photosynthetic efficiency, and promoting the transport of photosynthetic products [29]. Meanwhile, in recent years, BRs have also attracted extensive attention in alleviating extreme temperature stress, especially LT stress. For example, in a study by Xu et al., 0.1 mg L^−1^ 24-epbrassinolide (EBR) markedly alleviated the symptoms of wheat (*Triticum aestivum* L.) chilling injury by reducing the formation of malondialdehyde (MDA) [30]. In the study of Zhang et al., 0.1 μM EBR significantly improved the photosynthesis of cucumber (*Cucumis sativa* L.) by changing the activities of sedoheptulose-1,7-bisphosphatase, ribulose-1,5-biphosphate carboxylase/oxygenase, transketolase, and fructose-1,6-bisphosphate aldolase [31]. In the study of Ding et al., 0.3 mg L^−1^ EBR clearly prevented the oxidative stress of rice (*Oryza sativa* L.) seedlings by maintaining the levels of abscisic acid (ABA) and gibberellin 3 (GA_3_) [32]. In a study by Jiang et al., 0.1 mg L^−1^ BR application enhanced the LT tolerance of early seedlings in alfalfa (*Medicago sativa* L.) by regulating oligomeric proanthocyanidins content and phenylalanine ammonia-lyase activity [33]. In addition, coleoptile is one of the important organs to determine the stress tolerance, especially the LT tolerance of maize seedlings [34,35]. However, the effects of BRs on the coleoptile of maize seedlings, and the underlying physiological and molecular response mechanisms under LT stress, remain unclear.

Considering the sensitivity of coleoptiles to LT stress and the importance of exogenous BR applications in alleviating the cold injury of species, we assessed gene expression via RNA-sequencing (RNA-Seq) and profiled the metabolism of three sugars by physiological measurement in the coleoptiles of Zheng58 seedlings under four treatments, i.e., with or without 2.0 μM EBR application at 10 °C and 25 °C. At the same time, the gene interaction network was further constructed to reveal maize LT tolerance regulatory network. This work will provide a valuable reference for enhancing the LT tolerance of maize seedlings and breeding maize varieties with enhanced LT tolerance.

## 2. Results

### 2.1. Changes in Various Sugar Accumulation in Coleoptile Under LT Stress and EBR Stimulation

**Figure 1:**
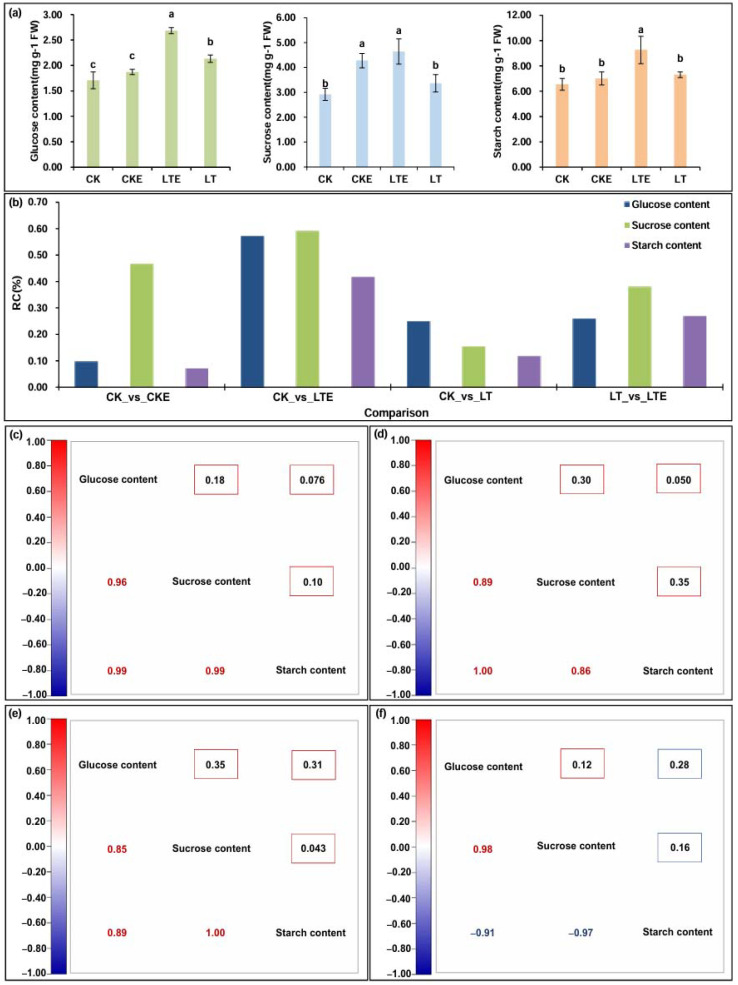
The rate changes (RCs) and relationships of three physiological traits in coleoptiles of Zheng58 seedlings under four treatments. CK: coleoptiles of Zheng58 seedlings treated with 0 μM 24-epibrassinolide (EBR) application at 25 °C normal temperature (CK); CKE: coleoptiles of Zheng58 seedlings treated with 2.0 μM 24-epibrassinolide (EBR) application at 25 °C normal temperature (CKE); LTE: coleoptiles of Zheng58 seedlings treated with 2.0 μM 24-epibrassinolide (EBR) application at 10 °C low temperature (LTE); LT: coleoptiles of Zheng58 seedlings treated with 0 μM 24-epibrassinolide (EBR) application at 10 °C low temperature (LT). (**a**) The contents of glucose, sucrose, and starch in coleoptiles under different treatments. (**b**) RCs of all traits in the CK_vs_CKE, CK_vs_LTE, CK_vs_LT, and LT_vs_LTE comparisons. (**c**) Pearson correlation coefficient among glucose, sucrose, and starch contents under CK treatment; the upper right is a *p*-value. (**d**) Pearson correlation coefficient analysis of glucose, sucrose, and starch content under CKE treatment; the upper right is a *p*-value. (**e**) Pearson correlation coefficient analysis of glucose, sucrose, and starch content under LTE treatment; the upper right is a *p*-value. (**f**) Pearson correlation coefficient analysis of glucose, sucrose, and starch content under LT treatment; the upper right is a *p*-value.

The pre-cultured coleoptiles of Zheng58 seedlings were exposed to four treatments for five days as follows: CK treatment, 25 °C normal temperature + 0 μM EBR application; CKE treatment, 25 °C normal temperature + 2.0 μM EBR application; LT treatment, 10 °C low-temperature + 0 μM EBR application; and LTE treatment, 10 °C low-temperature + 2.0 μM EBR application. The contents of glucose, sucrose, and starch in coleoptiles were then measured (Figure 1a). Interestingly, 10 °C LT stress caused a 24.9% increase in glucose content, a 15.3% increase in sucrose content, and 11.6% increase in starch content compared to CK control, respectively (Figure 1b), indicating that LT induced varied accumulation levels of different sugars, and their positive accumulation may be involved in the LT tolerance of maize. Compared to CK control, the contents of glucose and starch in Zheng58 coleoptiles increased slightly by 9.6% and 7.1% after CKE treatment, respectively, while their sucrose content increased significantly by 30% (Figure 1b). Moreover, a similar phenomenon of these sugar accumulations in Zheng58 coleoptiles was also observed under LTE treatment; this treatment increased glucose content (25.9%), sucrose level (37.9%), and starch formation (26.8%) compared with those that were cultured under LT stress, respectively (Figure 1b). It is thus speculated that a 2.0 μM EBR application promoted the formation and accumulation of various sugars in maize coleoptiles under different temperatural environments, and thus reduced the LT damage of maize seedlings.

### 2.2. Correlation Analysis

To more effectively clarify the correlation relationships among glucose, sucrose, and starch metabolisms in the coleoptiles of Zheng58 seedlings under all treatments, a correlation analysis of the three sugars was conducted. The results showed that there were similar correlation relationships among these sugar contents under CK, CKE, and LTE treatments (Figure 1c–e); namely, glucose content was positively correlated to sucrose content and starch content, and sucrose content was positively correlated with starch content, suggesting that the three sugars in maize coleoptiles exist in synergistic accumulations under the above treatments. By contrast, glucose content had a negative correlation to starch content, and sucrose content displayed a negative correlation to starch content in the coleoptile of Zheng58 seedlings under LT stress (Figure 1f), implying that these substances in maize coleoptiles compete to deposit more soluble sugars to better adapt to LT stress.

### 2.3. Overview of RNA-Seq Quality and DEGs Identification

To evaluate the LT stress and EBR-induced changes in the transcript profiles, the RNA-Seq from Zheng58 coleoptiles under four treatments (i.e., CK, CKE, LT, and LTE, with three biological replicates, in total 12 samples) was performed by the Illumina NovaSeq PE150 platform. An average of 52,134,440 clean reads were obtained from each sample and the Q30 value exceeded 93% (Table 1). After filtering low-quality reads, approximately 90.66–90.91% of clean reads were mapped to the Zea_mays.B73_RefGen_v4 reference genome. This suggested that the sequencing quality was high and could be used for the following analysis.

**Figure 2:**
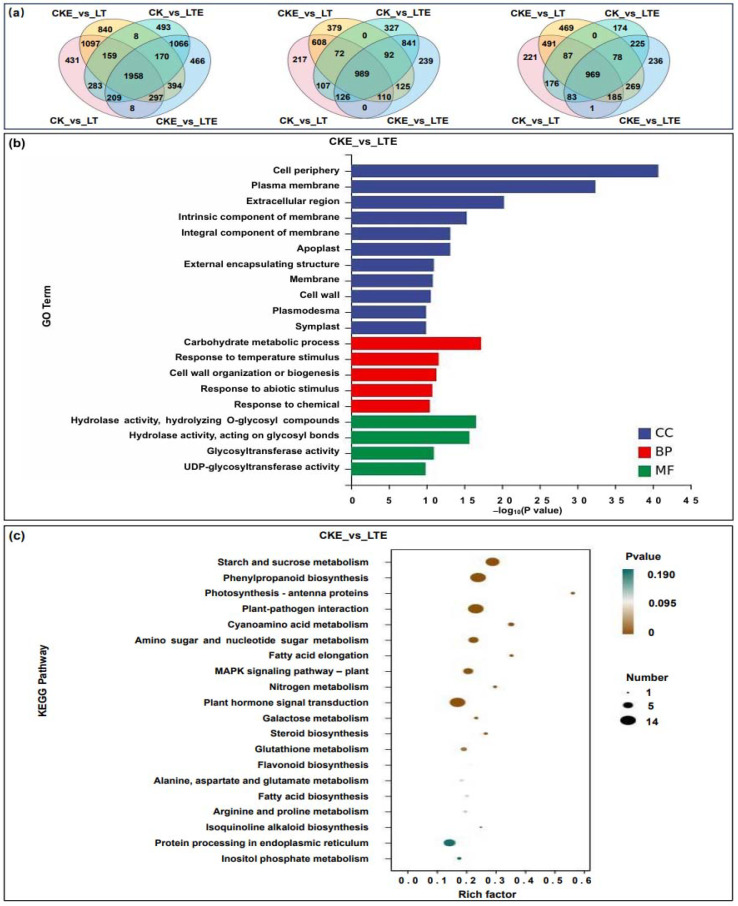
Differentially expressed genes (DEGs) were identified, GO annotation and top 20 KEGG pathways of DEGs were analyzed in the coleoptiles of Zheng58 seedlings under four treatments from transcriptomic data. CK: coleoptiles of Zheng58 seedlings treated with 0 μM EBR application at 25 °C normal temperature; LT: coleoptiles of Zheng58 seedlings treated with 0 μM EBR application at 10 °C low temperature; CKE: coleoptiles of Zheng58 seedlings treated with 2.0 μM EBR application at 25 °C normal temperature; LTE: coleoptiles of Zheng58 seedlings treated with 2.0 μM EBR application at 10 °C low temperature. (**a**) The total differentially expressed genes (DEGs), up-regulated differentially expressed genes (up-regulated DEGs) and down-regulated differentially expressed genes (down-regulated DEGs) in the CK_vs_CKE, CK_vs_LTE, CK_vs_LT, and LT_vs_LTE comparisons, respectively. (**b**) GO enrichment analysis of DEGs in CKE_vs_LTE comparison. (**c**) KEGG enrichment analysis of DEGs in the CKE_vs_LTE comparison.

Further, the DEGs from the different comparisons were identified based on the criteria of |log_2_ fold change (FC)| > 1, *p*-value < 0.05, and false discovery rate (FDR) < 0.05. In total, we identified 1958 DEGs that were common among the four comparisons, including CK_vs_LT, CK_vs_LTE, CKE_vs_LT, and CKE_vs_LTE, with 989 up-regulated DEGs and 969 down-regulated DEGs (Figure 2a). These data thus indicate that LT stress and EBR treatment significantly activated or inhibited expressions, which may play important roles in improving LT tolerance in maize coleoptiles. In addition, between the CK_vs_LT and CK_vs_LTE comparisons, there were a total of 283 DEGs, including 107 up-regulated DEGs and 176 down-regulated DEGs (Figure 2a). This finding implied that significantly down-regulated expressions of these common DEGs were conducive to the survival of maize seedlings under LT stress.

### 2.4. Functional Annotation and Enrichment Analysis of DEGs

To better clarify the functions of DEGs in Zheng58 coleoptiles under all treatments, the GO annotation and top 20 KEGG pathways were analyzed in the CCKE_vs_CLTE comparison. For GO annotation, the main categories were “plasma membrane” and “apoplast” among cellular components, “carbohydrate metabolic process” and “response to temperature stimulus” among biological processes, as well as “hydrolase activity”, “glycosyltransferase activity” and “UDP- glycosyltransferase activity” among molecular functions (Figure 2b). This showed that these DEGs play potential roles in protecting biofilm integrity, carbohydrate metabolism, and resistance response in Zheng58 coleoptiles. Likewise, the main categories of the KEGG pathways were “starch and sucrose metabolism”, “phenylpropanoid biosynthesis”, “photosynthesis-antenna proteins”, and “amino sugar and nucleotide sugar metabolism” (Figure 2c). This indicated that LT stress or exogenous EBR stimulation affected varied expressions of multiple genes involved in the above metabolic pathways to regulate the LT tolerance of maize coleoptiles.

It is well known that the phenylpropanoid biosynthesis is part of the shikimate pathway, synthesizing some bioactive secondary metabolites, including flavonoids, lignins, coumarins, and lignans, which play significant roles in plant growth, development, as well as responses to environmental stimuli, such as resistance against abiotic and biotic stresses [36].

### 2.5. DEGs Involved in Starch and Sucrose Metabolism

**Figure 3:**
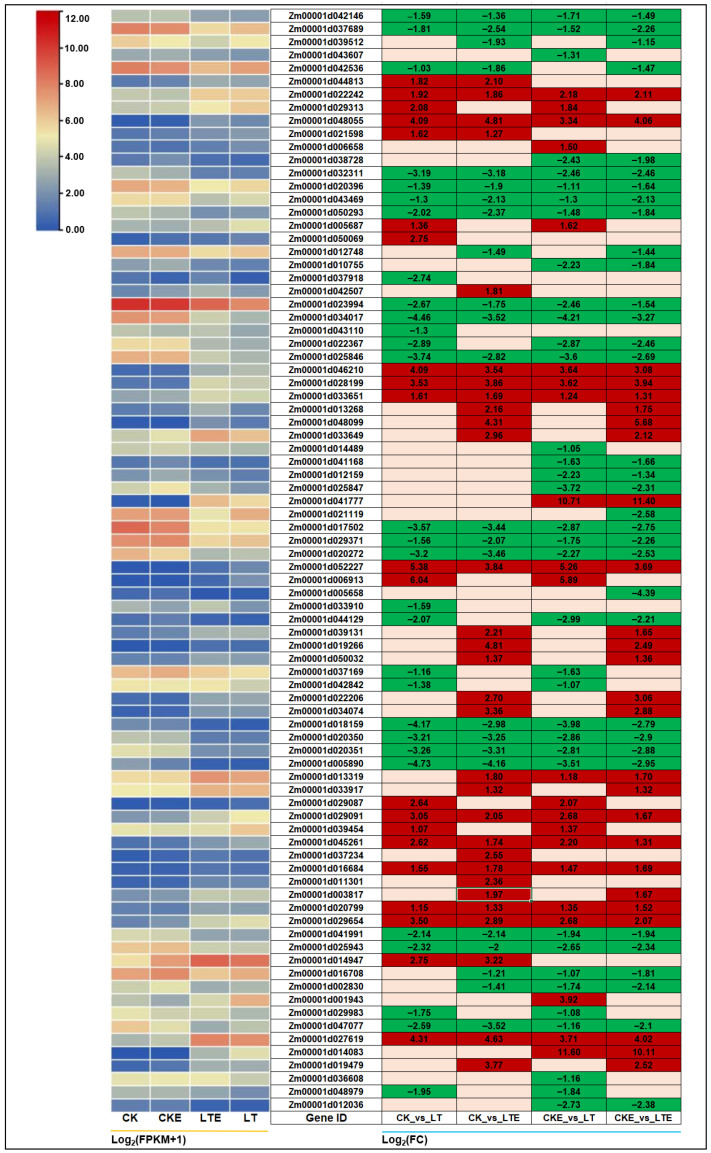
The expression profiles of differentially expressed genes (DEGs) involved in the sucrose and starch metabolism pathway in coleoptiles of Zheng58 seedlings were analyzed by RNA-sequencing (RNA-Seq). The left heatmap represents the expression level of differentially expressed genes under various treatments, shown as log_2_ (FPKM + 1), while the heatmap on the right represents the corresponding log2 (FC) of these genes among treatments compared to their controls. CK: coleoptiles of Zheng58 seedlings treated with 0 μM 24-epibrassinolide (EBR) application at 25 °C normal temperature; CKE: coleoptiles of Zheng58 seedlings treated with 2.0 μM 24-epibrassinolide (EBR) application at 25 °C normal temperature; LTE: coleoptiles of Zheng58 seedlings treated with 2.0 μM 24-epibrassinolide (EBR) application at 10 °C low temperature; LT: coleoptiles of Zheng58 seedlings treated with 0 μM 24-epibrassinolide (EBR) application at 10 °C low temperature. FPKM is the fragments per kilobase of transcript per million mapped reads; FC is the fold change.

Sugar is a major source of energy during the growth of living organisms and is known as the fuel of life, especially glucose. The production of endogenous sugar can improve the frost resistance of plants to reduce frost damage [36]. The changes in the expression levels of key sugar metabolism enzymes (SPS and SUS) and sucrose content may play significant roles in the cold response in maize [37]. For the starch and sucrose metabolism pathway, *Zm00001d029091* (sucrose synthase, SUS) was significantly up-regulated with expressions of 3.05-fold and 2.08-fold in the CK_vs_LT and CK_vs_LTE comparisons, respectively (Figure 3). In addition, two DEGs for glucan endo-1,3-beta-D-glucosidase (EGLC), including *Zm00001d048055* and *Zm00001d022242*, three DEGs associated with beta-glucosidase (BGLC), i.e., *Zm00001d046210*, *Zm00001d028199*, and *Zm00001d033651*, one starch synthase (glgA, *Zm00001d045261*) DEG, one isoamylase (treX, *Zm00001d020799*) DEG, and one beta-amylase (*Zm00001d027619*) DEG were significantly up-regulated, ranging approximately from 1.15 to 4.31-fold in the CK_vs_LT comparison and from 1.33 to 4.81-fold in the CK_vs_LTE comparison (Figure 3). However, the DEGs related to four alpha-amylases (AMY; including *Zm00001d018159*, *Zm00001d020350*, *Zm00001d020351*, and *Zm00001d005890*) and one beta-amylase (*Zm00001d047077*) showed significant down-regulation, varying approximately from –4.37 to –2.59-fold in the CK_vs_LT comparison and from –4.16~– to –2.98-fold in the CK_vs_LTE comparison (Figure 3). These findings suggest that multiple genes involved in starch and sucrose metabolism pathways display varied expression levels under different treatments, they may interact to regulate glucose, sucrose, and starch formation and accumulation in maize coleoptiles.

### 2.6. DEGs Involved in Photosynthesis

**Figure 4:**
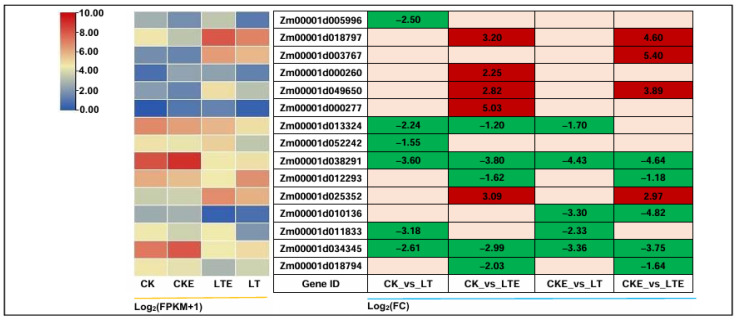
The expression profiles of differentially expressed genes (DEGs) involved in the photosynthesis pathway in coleoptiles of Zheng58 seedlings were analyzed by transcriptome sequencing. The left heatmap represents the expression level of differentially expressed genes under various treatments, shown as log2 (FPKM + 1), while the heatmap on the right represents the corresponding log2 (FC) of these genes among treatments compared to their controls. CK: coleoptiles of Zheng58 seedlings treated with 0 μM 24-epibrassinolide (EBR) application at 25 °C normal temperature; CKE: coleoptiles of Zheng58 seedlings treated with 2.0 μM 24-epibrassinolide (EBR) application at 25 °C normal temperature; LTE: coleoptiles of Zheng58 seedlings treated with 2.0 μM 24-epibrassinolide (EBR) application at 10 °C low temperature; LT: coleoptiles of Zheng58 seedlings treated with 0 μM 24-epibrassinolide (EBR) application at 10 °C low temperature.

Photosynthesis is an important physiological process of energy conversion in plants, which is very sensitive to LT [38]. It will be restrained under LT stress, affecting carbohydrate biosynthesis and plant growth and development [39]. In this study, we also tried to identify the DEGs for photosynthesis, a total of 15 DEGs were detected in the four comparisons (Figure 4). Specifically, a photosystem I subunit X (psaK, *Zm00001d018797*) had the largest positive expression level with 3.20-fold in the CK_vs_LTE comparison. The *Zm00001d038291* (petF, ferredoxin) and *Zm00001d034345* (petH, ferredoxin–NADP+ reductase) both belonging to system photosynthetic electron transport, were significantly down-regulated in all comparisons, with –3.60-fold and –2.61-fold in the CK_vs_LT comparison, respectively. Moreover, the other three DEGs, i.e., *Zm00001d018797* (psaK), *Zm00001d049650* (photosystem II oxygen-evolving enhancer protein 3, psbQ), and *Zm00001d025352* (petF) were significantly up-regulated in CK_vs_LTE and CKE_vs_LTE. The results indicate that when maize coleoptiles sensed LT stress, not only the genes encoding subunits of photosystem I changed, but genes encoding enzymes in photosystem II, including the photosynthetic electron transport chain, also changed significantly. We suspect that these changes in photosynthesis affect some metabolic processes of maize in response to LT.

### 2.7. Gene Interaction Network Construction

**Figure 5:**
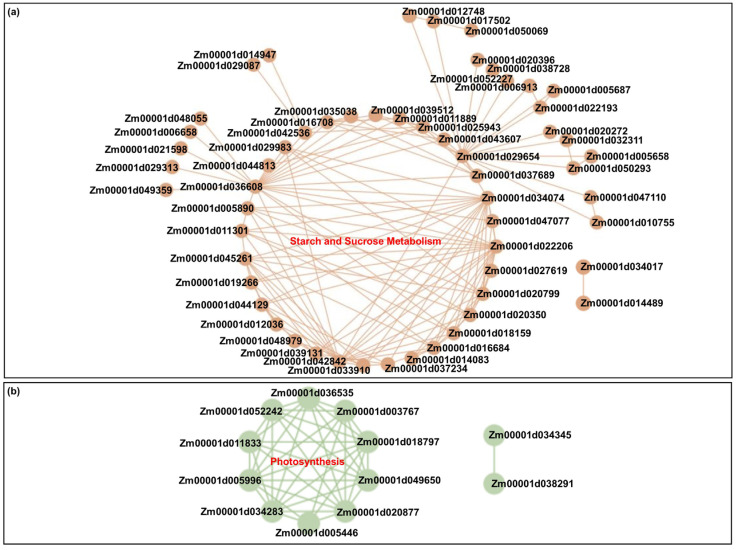
Gene Co-expression Networks between DEGs in sucrose and starch metabolism pathway and photosynthesis pathway in the coleoptiles of Zheng58 seedlings. Nodes represent proteins, connections between nodes represent interactions between proteins. (**a**) Gene co-expression network between DEGs in sucrose and starch metabolism pathway and photosynthesis pathway in the coleoptiles of Zheng58 seedlings. (**b**) Gene co-expression network between DEGs in photosynthesis pathway in the coleoptiles of Zheng58 seedlings.

The above identified 84 DEGs involved in starch and sucrose metabolism and 21 DEGs associated with photosynthesis among the four comparisons were used to construct their gene interaction network using Cytoscape 3.8.2 software (https://cytoscape.org/, accessed on 8 April 2024). Interestingly, there were 59 DEGs involved in starch and sucrose metabolism, many of which interacted with each other, forming a sugar signaling and metabolism regulatory network (Figure 5a). Meanwhile, we also found 12 DEGs associated with photosynthesis, which interacted with each, as shown in a photosynthesis network map (Figure 5b). Notably, there was no direct gene interaction between both pathways, which may be related to the weak photosynthesis of maize coleoptiles, or these DEGs may interact indirectly with DEGs or differentially expressed transcription factors (TFs) responsible for other crosstalk pathways.

### 2.8. Verification of RNA-Seq Data by Quantitative Reverse Transcriptase PCR (qRT-PCR)

**Figure 6:**
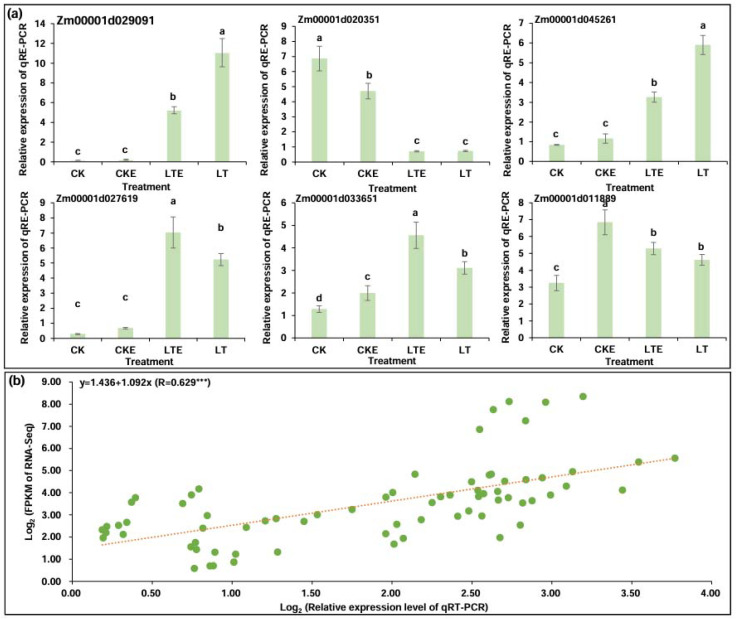
Quantitative reverse transcriptase PCR (qRT-PCR) analysis of six differentially expressed genes (DEGs) in coleoptiles of Zheng58 seedlings under four treatments. CK: coleoptiles of Zheng58 seedlings treated with 0 μM 24-epibrassinolide (EBR) application at 25 °C normal temperature; CKE: coleoptiles of Zheng58 seedlings treated with 2.0 μM 24-epibrassinolide (EBR) application at 25 °C normal temperature; LTE: coleoptiles of Zheng58 seedlings treated with 2.0 μM 24-epibrassinolide (EBR) application at 10 °C low temperature; LT: coleoptiles of Zheng58 seedlings treated with 0 μM 24-epibrassinolide (EBR) application at 10 °C low temperature. (**a**) The relative expression levels of six DEGs in coleoptiles of Zheng58 seedlings in the sucrose and starch metabolism under all treatments. (**b**) Correlation between qRT-PCR and RNA-Seq for selected six DEGs. *** represents significant difference (*p* < 0.001) by ANOVA.

To validate the reliability of RNA-seq data, DEGs *Zm00001d029091* (sucrose synthase 2), *Zm00001d020351* (alpha-amylase 1), *Zm00001d045261* (glgA, starch synthase I), *Zm00001d027619* (beta-amylase 2), *Zm00001d033651* (BGLC), and *Zm00001d011889* (hexokinase 3) were randomly selected for qRT-PCR analysis in Zheng58 coleoptiles under the four treatments (Figure 6a). The results of the expressions trends of qRT-PCR were consistent with the six DEGs in the RNA-seq dataset, and there was a good linear relationship between the expression level of the qRT-PCR and RNA-Seq dataset (y = 1.436 + 1.092x; R = 0.629 ***) (Figure 6b), reflecting the reliability of the RNA-Seq data.

## 3. Discussion

Maize output accounts for 40% of global food production [40,41]. As the world’s second largest corn producer, China contributes 23.5% of the global maize production, playing an important role in ensuring food security [42]. The spring corn area in North China is the main corn producing area in China, where cold injury has become one of the main climatic limiting factors affecting maize production [43]. Fortunately, currently cultivated maize has evolved a series of self-regulatory mechanisms to resist low temperature stress, such as sugar signaling and metabolic processes, though many questions remain. In this regard, this study investigated the changes of sugar levels in coleoptiles of Zheng58 seedlings under exogenous 2.0 μM EBR application at 25 °C (control) and 10 °C LT stress.

The coleoptile in maize is an important embryo organ that senses various stresses during seedling emergence, particularly LT stress; its elongation affects seedling emergence and morphological establishment [14,40]. In wheat, the elongated coleoptile can help the seed absorb moisture from deeper soil layers and ensure the seedling’s successful emergence, subsequently enhancing drought tolerance and yield in arid regions [44]. Increasingly, studies have reported that carbohydrates, proteins, and amino acids are important osmoregulatory substances that resist LT stress [45,46,47]. Soluble sugar, including glucose and sucrose as key osmotic and signaling molecules, are involved in plant response and adaptation to environmental stress, and promote starch hydrolysis [48]. In this study, 10 °C LT stress caused a 24.9% increase in glucose content, a 15.3% increase in sucrose levels, and an 11.6% increase in starch accumulation in Zheng58 coleoptiles (Figure 1b). At the same time, their relationships showed that starch accumulation was negatively correlated to glucose and sucrose formation at 10 °C LT stress (Figure 1f). These findings suggest that sugar biosynthesis and degradation in maize coleoptiles under LT stress are very complex, and their dynamic changes affect cold tolerance. Similar observations were made in *Jatropha curcas* L. seedlings, where 12 °C LT stress induced significant increases in β-amylase activity, sucrose phosphate synthase activity, and uridine diphosphate glucose phosphorylase, along with increases in glucose content, fructose level, galactinol accumulation, and raffinose formation [48].

BRs have been shown to improve the adaptability of most plants to biotic and abiotic stresses [49]. Although its alleviation effects on LT stress have been proven in wheat [30], rice [32], and tomato (*Solanum lycopersicum* L.) [50], little is known about the effects of this hormone on alleviating cold stress in early maize seedlings. In this study, we found that the accumulation of three carbohydrates in coleoptiles increased after spraying 2.0 μM EBR at a normal temperature of 25 °C, with glucose (9.6%), sucrose (12.3%), starch (7.1%) increase, respectively (Figure 1b); while these carbohydrate levels in coleoptiles significantly accumulated when they were treated with a 2.0 μM EBR spray at 10 °C stress pressure, with 24.9%, 15.3%, and 11.6% increase, compared to LT stress, respectively (Figure 1b). In peanut (*Arachis hypogaea* L.) seedlings, under 150 mM NaCl stress, an applicating of 0.1 μM EBR promoted the accumulation of soluble sugar and proline [51]. In the production of bell peppers (*Capsicum annuum* L.), a 0.05–0.20 μM EBR application ameliorated its heat stress by increasing some osmotic substances, including soluble sugars, starch content, proline content, and soluble proteins [52]. In Potato (*Solanum tuberosum* L.) tuber formation, the intercellular CO_2_ concentration, proline, and soluble sugar contents were decreased after 0.5 μM EBR pretreatment compared with plants under drought stress [53]. These data show that, like in other stress environments in various plants, exogenous applications of a suitable EBR concentration under LT stress in maize seedlings could improve the influence of adverse environments by increasing the sugar content and formation and deposition of other substances. Therefore, it is necessary to further identify the candidate genes involved in signaling and metabolism under LT stress and exogenous EBR induction to reveal the molecular mechanisms of LT tolerance in maize seedlings.

A total of 84 DEGs involved in starch and sucrose metabolism pathways were identified among the four comparisons from this study (Figure 3). It is well known that sucrose is the main form of sugar export [54]. Sucrose phosphate synthetase (SPS) and sucrose synthase (SUS) are key enzymes in the sucrose synthesis pathway [37,54,55]. During the germination of gramineous plants, glucose from starch stored in the endosperm is transported to the endosperm, where it is converted to sucrose by enzymes such as SPS [54]. SUS catalyzes the conversion of sucrose and nucleoside diphosphate into the corresponding nucleoside diphosphate-glucose and fructose and provides substrates for cellulose and starch biosynthesis: UDP-glucose and adenosine diphosphate-glucose (ADP-glucose) [56,57,58]. In this study, we detected two DEGs for SUS: *Zm00001d029091* showed a significant up-regulated expression in all four comparisons, and it had the largest FC value in the CK_vs_LT comparison (3.05-fold); and *Zm00001d029087* showed a significant up-regulated expression in the CK_vs_LT (2.64-fold) and the CKE_vs_LT (2.07-fold) comparisons. This suggests that LT stress and EBR stimulation activated the two SUS genes to promote sucrose production in maize coleoptiles. The glgA is the key enzyme directly involved in starch synthesis. Previously, Xu et al. [59] reported that *NtGBSS2* (a glgA) in *Nicotiana tabacum* L. was the core gene involved in its starch metabolism, and its overexpression significantly increased the content of starch and enhanced the plant’s drought tolerance. In our study, *Zm00001d045261* showed 2.61-fold, 1.74-fold, 2.20-fold, and 1.31-fold up-regulated expression in the four comparisons (Figure 3). We speculated that this starch synthase gene may play a crucial role in maize response to LT stress. The BGLCs are implicated in releasing glucose and aglycone molecules during fruit ripening [60]. Hashempour et al. [61] showed that BGLC could be involved in increasing frost tolerance in *Canarium album* L. plants. Conversely, we found 21 DEGs responsible for BGLCs, which showed varied expression profiles in all four comparisons, suggesting a regulatory complexity of LT tolerance involving BGLC genes in maize. For other DEGs in starch and sucrose metabolism pathways, their regulatory mechanisms in cold tolerance of maize remain to be further studied.

Photosynthesis, which is vital for plant growth and high productivity, is strongly influenced by BR signaling, and the photosynthetic rate is decreased under LT stress [62,63]. SPS plays a role as the limiting factor in the export of photoassimilates to sink tissues in leaves, and the activity of SPS is correlated with sugar accumulation in sink tissues [64]. Sugars, produced through photosynthesis, are the core of all organic compounds synthesized, and the maintenance of sugar partitioning between the different subcellular compartments and between cells is important in adjusting the photosynthesis performance and response to abiotic constraints [65]. Interestingly, in the current study, a total of 15 photosynthesis related DEGs were identified and showed significantly differential expression levels in the four comparisons (Figure 4). Whether there is a link between photosynthetic genes and sugar metabolism genes is unknown.

**Figure 7:**
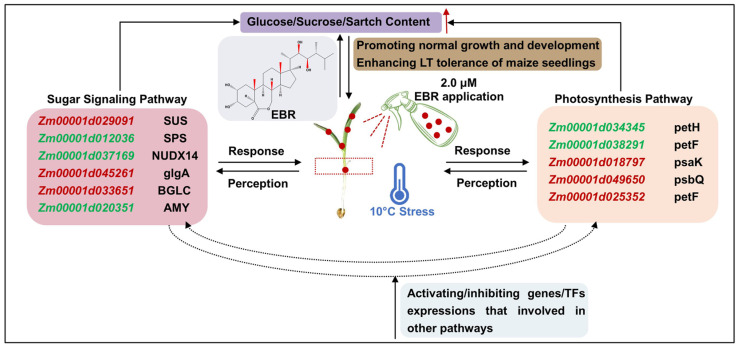
The model of molecular regulatory mechanism in response to low-temperature stress and exogenous 24-Epibrassinolide (EBR) stimulation of maize coleoptiles. The red font represents up-regulated DEGs, the green font down-regulated DEGs; the red upward arrow represents an increase of corresponding metabolite accumulation.

Based on our findings, and those reported in previous studies, we attempted to reveal the mechanism of sugar signaling, sugar metabolism, and photosynthetic changes of coleoptiles in maize seedlings under LT stress after exogenous 2.0 μM EBR application (Figure 7). Unfortunately, we did not find the direct interactions between sugar signaling and photosynthesis in our study. This is likely related to the selected organs/tissues and developmental period of maize; during seedling emergence, the photosynthetic capacity of the maize coleoptiles is very weak. We speculate that the corresponding DEGs for SPS and SUS are crucial nodes, which further form a potential link among photosynthesis, sugar signaling and sugar metabolism, and other unknown pathways, to determine LT tolerance of maize seedlings. Notably, a 2.0 μM EBR application significantly improved the tolerance of maize seedling to low temperatures. Therefore, this research provides new insights and ideas for cold resistance breeding in maize.

## 4. Materials and Methods

### 4.1. Experimental Materials and Treatments

The elite Zheng58 genotype, which is derived from the Reid heterotic group, is the female parent of the Zhengdan958 variety [40], and its seeds were produced at Longxi experimental station, Gansu, China (34.97° N, 104.40° E, 2074 m altitude). The seeds were disinfected in 0.5% (*v/v*) sodium hypochlorite solution for 10 min and washed with double-distilled water (ddH_2_O) to remove the residue of disinfectant, which were then washed with ddH_2_O for 24 h. Ten soaked seeds were sown in pots (height 17 cm, diameter 20 cm) containing vermiculite, and placed into an illumination incubator for five days at 25 °C in darkness, the corresponding etiolated seedlings were then placed into two illumination incubators at 25 °C and 10 °C, respectively, and were sprayed with 5.0 mL of 0 μM or 2.0 μM EBR solutions daily for five days in darkness. Overall, in total four treatments were performed in this study, as follows: CK treatment (0 μM EBR solution spray at 25 °C environment), LT treatment (0 μM EBR solution spray at 10 °C environment), CKE treatment (2.0 μM EBR solution spray at 25 °C environment), and LTE treatment (2.0 μM EBR solution spray at 10 °C environment), with three biological replicates. The corresponding coleoptiles of all treated Zheng58 seedlings were collected for physiological measurement, RNA-Seq analysis, and qRT-PCR gene verification in following studies.

### 4.2. Physiological Metabolisms Measurements

Glucose, sucrose, and starch content of coleoptiles in Zheng58 seedlings under the above described four treatments were determined according to the method described by Huang et al. [66]. Namely, 0.2 g fresh coleoptiles were placed in a 10 mL centrifuge tube, and 5 mL of 80% (*v/v*) ethanol solution were added to incubate for 30 min in a 80 °C water bath; when the temperature of the centrifuge tube dropped to room temperature to centrifuge 10 min at 3500 rpm, and the supernatant was collected; the preceding steps were repeated twice. Subsequently, an enzyme-labeling method was used to measure the glucose content at 460 nm, resorcinol method was used to determine the sucrose content at 485 nm, and anthrone–sulfuric acid method was used to assay the starch content at 620 nm, respectively.

### 4.3. Statistical Analyses

For all physiological metabolisms of Zheng58 coleoptiles under four treatments, their rate changes (RCs) from different comparisons (CCK_vs_CCKE, CCK_vs_CLTE, CCK_vs_CLT, and CLT_vs_CLTE) were calculated as follows [14]:

RC_(CCK_vs_CCKE)_ = (T_CCKE-i_ − T_CCK–i_)/T_CCK–i_ × 100% (1)

RC_(CCK_vs_CLTE)_ = (T_CLTE–i_ − T_CCK–i_)/T_CCK–i_ × 100% (2)

RC_(CCK_vs_CLT)_ = (T_CLT–i_ − T_CCK–i_)/T_CCK–i_ × 100% (3)

RC_(CLT_vs_CLTE)_ = (T_CLTE–i_ − T_CLT–i_)/T_CLT–i_ × 100% (4)

where T_CCK-i_, T_CCKE-i_, T_CLT-i_, and T_CLTE-i_ was the *i*-th physiological metabolism of single maize genotype under CK, CKE, LT, and LTE treatment, respectively. Pearson correlation was performed using Origin 2021 (v. 21.0, OriginPro, USA; https://www.genescloud.cn; accessed on 20 March 2024). For all traits of the Zheng58 coleoptiles under four treatments, one-way ANOVA was performed using IBM-SPSS Statistics v.20.0 software (SPSS, Chicago, IL, USA; https://www.Ibm.com/products/spss-statistics; accessed on 16 March 2024).

### 4.4. RNA-Seq Analysis, DEGs Identification, GO and KEGG Enrichment Analysis

The coleoptile samples under the CK, CKE, LT, and LTE treatments in Zheng58 were collected in three replicates to perform RNA-Seq using the BGI DNBSEQ-T7 system by Shanghai Personal Biotechnology Co., Ltd. China. After sequencing, the clean reads were obtained and aligned to the *Zea mays* B73_v4 reference genome (ftp://ftp.ensemblgenomes.org/pub/plants/release-6/fasta/zea_mays/dna/; accessed on 29 March 2024) using HISAT2 software (http://ccb.jhu.edu/software/hisat2; accessed on 29 March 2024). The data were analyzed using HTSeq software (http://htseq.readthedocs.io/en/release_0.9.1/; accessed on 30 March 2023) based on the readcount data obtained from expression profiling and the fragments per kilobase per million mapped (FPKM) values were calculated. The DEGs were defined using the criteria: |log_2_ FC| > 1, *p*-value < 0.05, and FDR < 0.05 via DESeq2 package (http://bioconductor.org/packages/release/bioc/html/DESeq.html; accessed on 29 August 2024). For identified DEGs, GO enrichment analysis was performed using AmiGO 2 database (http://amigo.geneontology.org/amigo/; accessed on 29 August 2024); KEGG analysis was performed using KEGG database (https://www.kegg.jp/kegg/; accessed on 29 August 2024).

### 4.5. Gene Interaction Network Mapping

For the DEGs for starch and sucrose metabolism pathway and photosynthesis pathway, their interaction network mapping was constructed using Cytoscape v.3.7.1 (https://cytoscape.org/; accessed on 8 April 2024).

### 4.6. qRT-PCR Validation

To ensure the reliability of the RNA-Seq results, six random DEGs were selected for gene validation by qRT-PCR. Single-stranded cDNA was obtained using PrimeScript™ 1st stand cDNA synthesis Kit (Takara, Japan). Gene special primers were designed using the Primer3web v.4.1.0 (https://primer3.ut.ee/; accessed on 16 April 2024). The LightCycler480II fluorescent quantitative PCR instrument (Roche, Germany) was used for qRT-PCR analysis. The *ZmACT-1* was used as the internal control for normalization [40]. There were three replicates for gene relative expression analysis, and the relative gene expression level was estimated by the 2−ΔΔCT method [40]. The analysis of variance (ANOVA) of relative expression levels was performed using IBM-SPSS Statistics v.20.0 software (SPSS, Chicago, IL, USA; https://www.Ibm.com/products/spss-statistics; accessed on 16 March 2024).

## 5. Conclusions

In conclusion, a 2.0 μM exogenous EBR application promoted the accumulation of various carbohydrates (i.e., glucose, sucrose, and starch) by activating/inhibiting multiple genes expressions involved in starch and sucrose metabolism pathways in maize coleoptiles; at the same time, we speculate that some genes regulating photosynthesis also indirectly interact with related starch and sucrose metabolism genes. These increased sugars may act as signaling molecules and osmoregulators to enhance LT tolerance in maize seedlings. Therefore, these findings enhance our understanding of how an exogenous EBR application improves maize LT tolerance.

## Figures and Tables

**Table 1:** Statistics of sequence reads and mapping efficiency.

Samples	Raw Reads	Clean Reads	Mapped Reads	Mapped Ratio (%)	Q30 (%)
CK	47,611,863	49,158,055	42,551,020	90.75	93.80
CKE	49,383,388	51,624,310	44,099,804	90.66	93.85
LT	50,478,915	52,744,770	45,205,806	90.91	93.73
LTE	55,010,625	55,010,625	49,190,630	90.87	93.69

## Data Availability

Data are contained within the article.
